# Book Notices

**Published:** 1869-10

**Authors:** 


The Membrana Tympani in Health and Disease. Illustrated by 24 chromo-lithographs. Clinical Contributions to the Diagnosis and Treatment of Diseases of the Ear, with Supplement. By Dr. ADAM POLITZER, of the University of Vienna. Translated by A. MATHEWSON, M.D., and H. G. NEWTON, M.D., Assistant-Surgeons of the Brooklyn Eye and Ear Hospital; Members of the Amer. Ophthalmological and Otol. Society. New York: WM. WOOD & Co., 61 Walker Street 1869.

This is a volume of 183 pages, octavo, neatly bound, printed on good type and paper, with beautiful illustrations. The subject of which it treats is a very important one, yet but little understood by the general practitioner. We commend the work as one of real practical value. Price $2.50.

---

The Science and Art of Surgery; being a Treatise on Surgical Injuries, Diseases, and Operations. By JOHN ERIC ERICHSEN, Senior Surgeon to University College Hospital, and Holme Professor of Clinical Surgery in University College, London. From the fifth enlarged and carefully revised London edition. Illustrated with 630 engravings on wood. With additions. By JOHN ASHHURST, JR., A.M., M.D., Vice-President of the Philadelphia Pathological Society; Fellow of the College of Physicians; Member of the Academy of Natural Sciences; Surgeon to the Episcopal Hospital, etc., etc. Philadelphia: HENRY C. LEA. 1869.

This is a large-sized octavo volume of 1228 printed pages; and, as its title-page imports, it is a thoroughly revised and enlarged edition of Erichsen's well-known work on surgery. The high reptutation gained by former editions will be increased by this. It is a good, practical work, both for study and reference. Price, bound in cloth $7.50, in sheep $8.50. For sale by S. C. Griggs & Co., 117 and 119 State Street, Chicago.

---

A Guide-Book of Florida and the South, for Tourists, Invalids, and Emigrants, with a Map of the St. John River. By Daniel G. Brinton, A.M., M.D. Philadelphia: George MacLean, 719 Sansom Street. 1869.

This is a duodecimo volume of 136 pages, written in a pleasant style, and presenting a great variety of very valuable information, for the use of such as desire to visit the South, and especially Florida. It is also a very useful volume for physicians who wish to be prepared to give proper directions to such of their patients as may wish to resort to Florida, for the improvement of health. Aside from a slight commendation of the use of alcoholic liquors, in the last section, that we wish the author had omitted, we freely recommend the work, both to the profession and the public. Price $1.

